# The interpretation of behavior-model correlations in unidentified cognitive models

**DOI:** 10.3758/s13423-020-01783-y

**Published:** 2020-08-06

**Authors:** Leendert van Maanen, Steven Miletić

**Affiliations:** 1 Department of Experimental Psychology, Utrecht University, Utrecht, Netherlands; 2 University of Amsterdam, Amsterdam, Netherlands

**Keywords:** Decision making, Math modeling, Signal detection theory, Reinforcement learning

## Abstract

The rise of computational modeling in the past decade has led to a substantial increase in the number of papers that report parameter estimates of computational cognitive models. A common application of computational cognitive models is to quantify individual differences in behavior by estimating how these are expressed in differences in parameters. For these inferences to hold, models need to be identified, meaning that one set of parameters is most likely, given the behavior under consideration. For many models, model identification can be achieved up to a scaling constraint, which means that under the assumption that one parameter has a specific value, all remaining parameters are identified. In the current note, we argue that this scaling constraint implies a strong assumption about the cognitive process that the model is intended to explain, and warn against an overinterpretation of the associative relations found in this way. We will illustrate these points using signal detection theory, reinforcement learning models, and the linear ballistic accumulator model, and provide suggestions for a clearer interpretation of modeling results.

**Electronic supplementary material:**

The online version of this article (10.3758/s13423-020-01783-y) contains supplementary material, which is available to authorized users.

The rise of computational modeling in the past decade has led to a substantial increase in the number of papers that report parameter estimates of computational cognitive models (Lebreton, Bavard, Daunizeau, & Palminteri, 2019; Palminteri, Wyart, & Koechlin, 2017; Tran, van Maanen, Matzke, & Heathcote, submitted). The general goal of such models is to capture theories of cognitive functioning in mathematical or computational form. For example, signal detection theory (SDT; Green & Swets, 1966) has sought to understand the detection of signals in a noisy environment as a probabilistic process, in which the disambiguation of stimuli and no stimuli depends on the strength of the (internal representation of the) signal and a detection criterion. Reinforcement learning (Sutton & Barto, 2018) models are aimed at understanding the learning processes involved when people learn from the outcome of repeated choices. A third prominent class of computational cognitive models—sequential sampling models or evidence accumulation models—theorizes that choice behavior is the result of a gradual accumulation of evidence for the choice alternatives, until a certain criterion or threshold value is reached. This idea is quantified in mathematical models such as the diffusion decision model (Ratcliff, 1978; Ratcliff & McKoon, 2008) or the linear ballistic accumulator model (LBA; S. D. Brown & Heathcote, 2008). All these models have in common is that in order to understand behavior, parameters are estimated that are hypothesized to quantify aspects of cognitive processing (Turner, Forstmann, Love, Palmeri, & van Maanen, 2017; Wilson & Collins, 2019).

A common application of computational cognitive models is to quantify individual differences in behavior by estimating how these are expressed in differences in parameters (Lebreton et al., 2019). These are then linked to pertinent neurophysiological, psychological, or physical factors to understand how those factors give rise to different behavioral patterns (Mulder, van Maanen, & Forstmann, 2014; O’Reilly & Mars, 2011; Turner et al., 2017). For example, in one of the earlier examples of this approach, Forstmann et al. (2008) showed that individual differences in one parameter of the LBA model correlated with individual differences in percentage signal change of the blood-oxygen-level-dependent (BOLD) response in brain areas associated with delaying an action (particularly the striatum). In this way, these researchers showed that the LBA model captured a property of behavior that in the brain is associated with striatal activation.

It is important that the variables in an individual differences analysis are well understood. For example, Lebreton et al. (2019) argued that the assumptions in the neurophysiological measurements (specifically, BOLD) could influence both the strength and direction of a correlation between BOLD responses and some behavioral or model-based variable. In particular, they argued that the interpretation of interindividual differences in BOLD differs according to the underlying neural coding principle that is assumed by the researcher. Different coding principles lead to different choices in the analysis of the behavioral variable (specifically, *z* scoring or not), which in turn influences the scale of the behavioral measurement (e.g., Louie & Glimcher, 2012; Poldrack, 2015). These theoretical considerations affect the analysis choice of standardizing (*z* scoring) the behavioral variable or not, which in turn influences the scale of the behavioral measurement.

Here, we argue that  in addition scaling assumptions in the cognitive model parameters affect the observed correlations. Specifically, the cognitive model parameters should be identified (Moran, 2016). Model identification entails that the observed data are implied most strongly by a unique set of parameter values. That is to say, if one set of observed data is equally likely to have been generated by two or more sets of parameters (and no other, unique, set of parameters is *more* likely to have generated the data), a model is unidentifiable and the true parameters may not be known. For many models, including SDT, the RL, and LBA models introduced above, model identification can be achieved by enforcing an equality constraint, which means that under the assumption that one parameter has a specific value, all remaining parameters are identified.

Roughly a decade ago, Donkin, Brown, and Heathcote (2009) already noted some of the issues arising from this equality constraint—from now on referred to as *scaling constraint* as is typical in the mathematical psychology domain—in the context of evidence accumulator models. In particular, Donkin et al. showed that the magnitude of the scaling constraint acts as a multiplier for the remaining parameters. For that reason, a constant scaling factor is necessary to estimate the remaining parameters. Importantly, Donkin et al. (2009) warned against a potential overconstraint, when researchers routinely apply the scaling constraint *in every condition.* In cases where other (nonscaling) parameters are assumed constant across conditions—for example because it is assumed that these parameters do not systematically differ across conditions—this can lead to an unnecessarily strict model, compromising the goodness of fit. Rather, when nonscaling parameters are held constant across conditions, the scaling constraint should be fixed to a constant in one condition only, but for every participant.

In an extension of this work, in the current theoretical note, we show that constraining the scaling parameter *across participants* affects the outcome and interpretation of individual difference analyses based on the parameter estimates.

We argue that the scaling constraint implies a strong assumption about the cognitive process that the model is intended to explain, and warn against an overinterpretation of the associative relations found in this way. We will illustrate these points first using SDT (Green & Swets, 1966) and then using an RL model (Sutton & Barto, 2018). Finally, we will show the consequences of different scaling constraints in a reanalysis of data from an earlier study. In this study, LBA model parameters were associated with behavior in a secondary task (Miletić & van Maanen, 2019). The reanalysis reveals what inferences we might have drawn under different scaling assumptions.

The considerations that we will discuss in the current theoretical note emphasize that it is important to keep track of what the parameters of a cognitive model represent. Specifically, many parameters can be expressed relative to each other. Fitting the model while keeping one parameter constant for scaling purposes may change the parameter values, but not the relationship between them. An easy way to explicitly address this is by including the units of parameters whenever possible. By analogy, the fuel efficiency of a car is typically not expressed in a volumetric measure (e.g., liters), nor in a distance that can be travelled (e.g., in kilometers), but in the ratio of these two (l/km). Expressing the fuel efficiency using the ratio of units reminds car manufacturers and automotive journalists about the meaning of the quantity. This is especially useful when sometimes the inverse of this ratio is preferred (e.g., in the U.S., miles/gallon is often used). Although this example does not involve fixing a parameter to a constant (e.g., always assume that only 1 km has been travelled), it does illustrate that parameters are often interpreted relative to each other, which is also our perspective when it comes to parameters of computational cognitive models.

## Signal detection theory

**Fig. 1:**
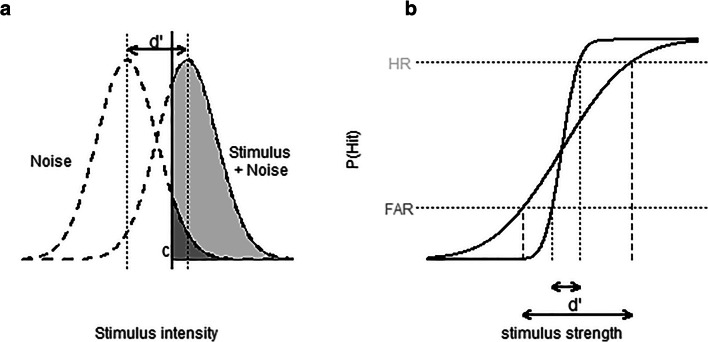
**a** Signal detection theory assumes that discriminating between the presence and absence of a signal depends on the discriminability of the signal (*d'*) as well as an internal criterion (*c*). Estimating these parameters depends on the hit rate (the light grey area under the stimulus curve) and the false-alarm rate (the dark grey area under the noise curve). **b** The mapping from the observed hit rate (HR) and false-alarm rate (FAR) onto *d'* for two different standard deviations (*s*) of the cumulative normal distributions, showing that *d'* scales with *s*

SDT (Green & Swets, 1966; Macmillan & Creelman, 2005) aims to understand the detection of signals in a noisy environment as a process in which the disambiguation of stimuli and no stimuli depends on the strength of the signal (typically referred to as *d'*) and a detection criterion (*c*). A typical assumption is that the stimulus is added to the noise distribution, resulting in the stimulus + noise distribution (see Fig. 1a). Based on the observed data from a detection experiment, *d'* and *c* can be estimated. In particular, the proportions of successful detections of the stimuli (hit rate [HR]), and incorrect reports of detection (false-alarm rate [FAR]) lead to $\hat{\mathrm{d}^{\prime }}=\mathrm{Z}\left(\mathrm{HR}\right)-\mathrm{Z}\left(\mathrm{FAR}\right)$, and $\hat{\mathrm{c}}=\left(\mathrm{Z}\left(\mathrm{HR}\right)+\mathrm{Z}\left(\mathrm{FAR}\right)\right)/2$, with *Z*(.) as the inverse standard normal cumulative distribution function. Because the standard normal distribution has a standard deviation of 1 by definition, SDT models are typically constrained to this value, but this is essentially an arbitrary choice. This can be illustrated by showing the relationship between *d'* and *c*, and the standard deviation *s* of the noise distribution. Figure 1b shows two cumulative probability density functions of the stimulus + noise distribution, mapping the observed stimulus strengths (on the *x*-axis) onto the cumulative probability of a hit (on the *y*-axis). The CDFs differ with respect to their assumed standard deviations. Based on the HR and FAR that are measured, the *d'* that is computed differs between the two distributions. The criterion value *c* shifts in a similar way as a function of *s*.

One common application of SDT is to understand individual differences in the criterion parameter (e.g., de Lange, Rahnev, Donner, & Lau, 2013; Kaneko & Sakai, 2015; Rahnev, Lau, & de Lange, 2011). It is easy to see how a different scaling assumption (e.g., *d'* = 1) would lead to a different pattern of individual differences. To illustrate this, we simulated data from 20 participants with varying criterions (200 trials each). Half of the trials consisted of noise trials in which we sampled from a normal distribution *N*(0, *s*). If the sample exceeded the criterion *c,* the trial was scored as a false alarm; otherwise, it was scored as a correct rejection. The other half of the trials were target trials in which we sampled from *N*(*d'*, *s*). If the sample exceeded the criterion, it was scored as a hit; otherwise, it was scored as a miss. For every participant, the parameters of the simulation were drawn from the following distributions, with *F* as a numeric factor ranging from −1 to 1:

$$ {\displaystyle \begin{array}{c}\mathrm{c}\sim \mathrm{U}\left(\mathrm{F}-0.1,\mathrm{F}+0.1\right),\\ {}{\mathrm{d}}^{\prime}\sim \mathrm{U}\left(0.9,1.1\right),\\ {}\mathrm{s}\sim \mathrm{U}\left(0.7,1.3\right).\end{array}} $$

We used a standard one-dimensional optimizer to obtain the standard deviation of the target and noise distribution that would satisfy this constraint. Note that this is potentially an overconstraint (Donkin et al., 2009), since we assume (following standard SDT) that the target and noise distributions have the same standard deviation.

**Fig. 2:**
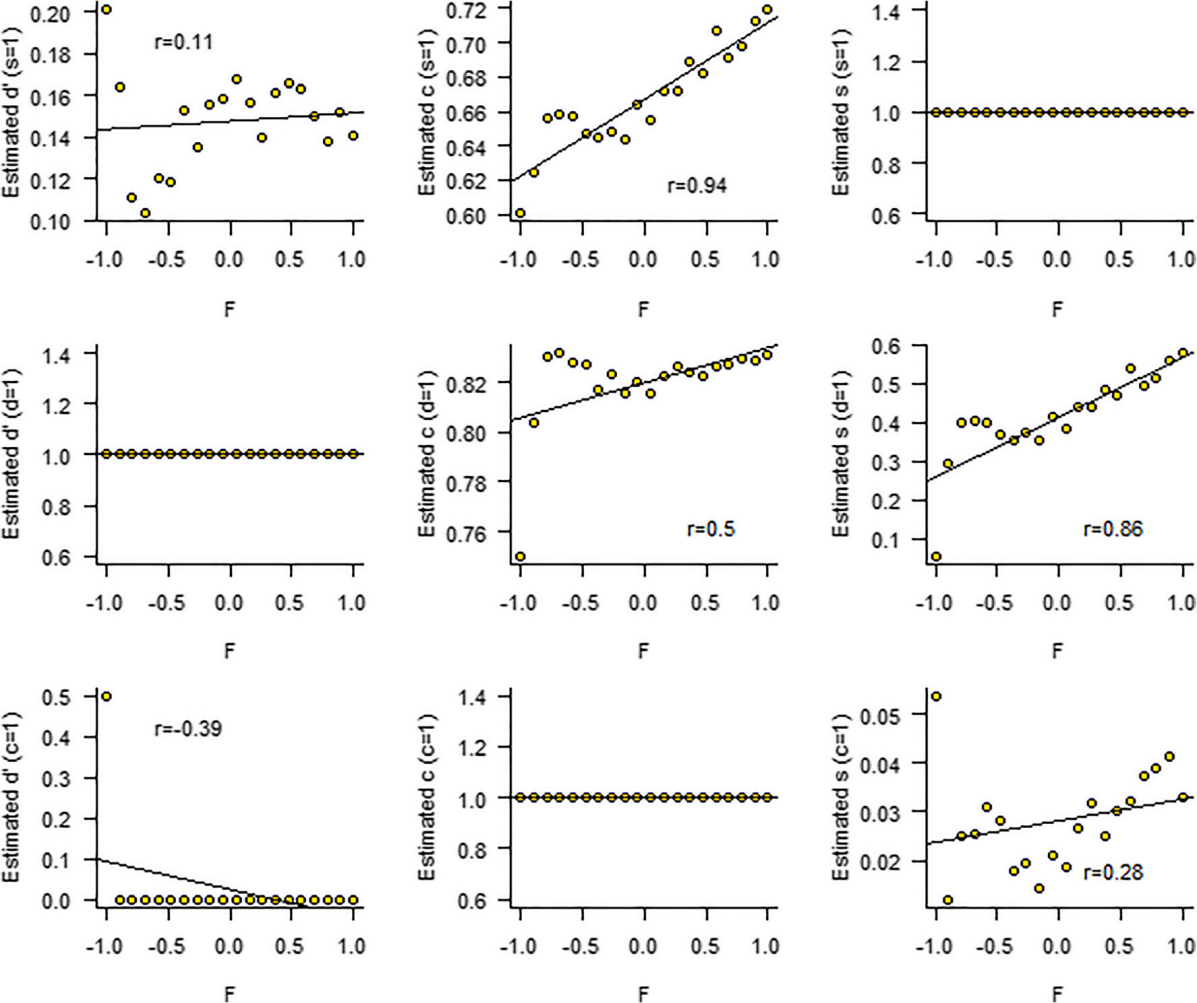
The correlations between an underlying factor *F* and the parameters of a signal detection theory model (SDT; Green & Swets, 1966), under different scaling constraints. Every row represents an attempt to estimate parameters of a scenario where *F* correlates with criterion *c*. The top row represents the standard practice that assumes that the standard deviation *s* is scaled to 1. The middle row assumes that *d'* is scaled to 1. The bottom row assumes that the criterion *c* is scaled to 1. Each attempt results in different conclusions about the relationships between the model parameters and factor *F*

The top panels of Fig. 2 display the default result (the standard deviation of the standard normal distribution is 1 by definition). In this scenario, with this particular data set, we find a strong interindividual correlation between factor *F* and criterion *c*, which is in line with the data-generating model. However, had we assumed *d'* was scaled to 1, we might have concluded that *F* correlates most strongly with standard deviation *s*, and to a lesser extent with criterion *c*. Finally, under the potential constraint *c* = 1, we might have concluded there is a weak but negative correlation between *F* and *d',* and an even weaker correlation between *F* and *s*.

Making the scaling constraint explicit in the parametrization of the model helps to interpret the observed correlations. In the simulation presented in Fig. 2, the top row represents a model where the parameters are scaled to the standard deviation of the stimulus + noise distribution (the default in SDT). This would mean that the underlying factor *F* correlates with the criterion *c per one standard deviation of the stimulus + noise distribution*, or that *F* correlates with *c/s*. Similarly, the middle row in Fig. 2 shows that *F* correlates with *s/d'*, or the standard deviation per one “signal unit.” The bottom row of Fig. 2 shows a negative correlation between *F* and *d'/c*. This might be interpreted as a correlation between *F* and the signal/criterion ratio.

The simulation of an SDT study shows that even in very simple experimental designs, conclusions about behavior-model correlations may be ambiguous. The ambiguity in conclusions in SDT generalizes to all other models that are identified up to a scaling constraint. In the next section, we specify how the implicit scaling of reward in RL models leads to the same issues.

## Reinforcement learning

RL models (Sutton & Barto, 2018) are typically applied to understand behavior in tasks in which the participant makes repeated decisions between multiple-choice alternatives, and each choice alternative gives a probabilistic reward. It is assumed that by trial and error, the participant discovers which choice alternative leads to the overall largest reward and should be preferred.

RL models consist of two parts: A learning rule and a choice rule. The learning rule determines how an internal representation of subjective value associated with each choice option changes based on feedback. Learning rules typically take the shape of

$$ {V}_{i,t+1}={V}_{i,t}+\alpha \left({r}_t-{V}_{i,t}\right), $$

where *V* is the subjective value associated with choice option *i* on trial *t*, *r* is the reward (feedback) received for a choice, and *α* a free parameter called the learning rate, which governs the volatility of *V* (Behrens, Woolrich, Walton, & Rushworth, 2007). The difference between the feedback and internal value, *r*_*t*_ − *V*_*i*, *t*_, is also known as the reward prediction error.

In addition to the learning rule, RL models are characterized by a choice rule. The choice rule determines the mapping between internal representations of value to choice probabilities. The choice rule is typically soft-max:

$$ {P}_i=\frac{\exp \beta {V}_i}{\sum_n^N\exp \beta {V}_n}, $$

where *β* is a free parameter called the inverse temperature. Soft-max assumes that the probability of choosing option *i* increases monotonically with *V*_*i*_ (assuming *V*_*j* ≠ *i*_ are constant) according to a sigmoidal function. The inverse temperature parameter *β* controls the steepness of the slope of the sigmoid, with higher *β* values leading to steeper slopes, and consequently more deterministic choices. This parameter is often interpreted in terms of the exploration–exploitation trade-off (e.g., Daw, O’Doherty, Dayan, Seymour, & Dolan, 2006).

In this model, *β* is only identified because of an implicit assumption formalized in the reward prediction error *r*_*t*_ − *V*_*i*, *t*_. The externally presented feedback *r* (e.g., “100 points” in a hypothetical experiment) is transformed into an internal representation of this feedback. The implicit assumption is that this process takes the shape of an identity function, *f*(*r*) = *r*. Alternative potential specifications involve a proportional mapping of external feedback to the internal representation, and even include nonlinear functions, such as a power law *f*(*r*) = *r*^*θ*^ to allow for diminishing marginal returns (with *θ* < 1). This specification is common in dominant economic decision-making theories such as utility theory and prospect theory (see, e.g., Ahn, Busemeyer, Wagenmakers, & Stout, 2008; Scholl, Kolling, Nelissen, Browning, et al., 2017a; Scholl, Kolling, Nelissen, Stagg, et al., 2017b; Scholl et al., 2015; Steingroever, Wetzels, & Wagenmakers, 2014, for examples of reinforcement learning models that adhere to utility and prospect theory).

Here, we address the simplest implicit assumption, but it is important to stress that this principle applies to more complex nonlinear mappings as well. If we assume the mapping function is linear, it need not be an *identity* function. A reward of, say, 100 points need not be equally valuable for all participants (V. M. Brown et al., 2018; Huys, Pizzagalli, Bogdan, & Dayan, 2013). Furthermore, the subjective value of an externally presented (constant) reward could also dynamically change during an experiment due to changing motivational factors (Berridge, 2012; Zhang, Berridge, Tindell, Smith, & Aldridge, 2009). We can explicate the parameter that governs the weighting of externally presented feedback to an internal representation of this feedback: *f*(*r*_*t*_) = *γr*_*t*_. In this case, the learning rule becomes

$$ {V}_{i,t+1}={V}_{i,t}+\alpha \left(\gamma {r}_t-{V}_{i,t}\right)=\left(1-\alpha \right){V}_{i,t}+\alpha \gamma {r}_t. $$

Dividing both sides of the equation by *γ* gives

$$ \frac{V_{i,t+1}}{\gamma }=\left(1-\alpha \right)\frac{V_{i,t}}{\gamma }+\alpha {r}_t, $$

highlighting that in essence, the *γ* parameter scales the internal representations *V*. Incorporating the scaled *V *in the soft-max choice rule illustrates that the RL model is unidentified unless either *β* or *γ* is assumed constant:

$$ {P}_i=\frac{\exp \frac{\beta {V}_i}{\gamma }}{\sum_n^N\exp \frac{\beta {V}_n}{\gamma }}. $$

Turning back to the individual differences focus of our note, the consequence is that any factor that correlates with *β* (under the assumption that *γ* = 1) is also associated with *γ* (under the assumption that *β* = 1). Put differently, the correlation is really with the ratio *β*/*γ*, which could be interpreted as a correlation with the inverse temperature per unit of weighted reward.

It is interesting to note that the learning rate parameter *α* remains identified irrespective of the choice of scaling constraint. It follows that if a researcher is interested in individual differences in both *β* and *γ*, scaling *α* will not help, as this will not identify the remaining parameters.

## Example application using linear ballistic accumulator

Most evidence accumulation models, including the well-known diffusion decision model (Ratcliff, 1978) and the LBA (S. D. Brown & Heathcote, 2008), share the issues sketched for SDT and RL. Supplementary Materials 1 and 2 present similar simulated scenarios as for SDT, for LBA, and DDM, respectively, illustrating that the observed associations depend on the scaling constraints in these models. Here, we present a reanalysis of previously published data using the LBA model to study how different scaling assumptions can potentially alter conclusions in a real data set. The data come from a recent paper of ours, that studied the relationship between decision-making and temporal reproduction (Miletić & van Maanen, 2019). In Experiment 1, we correlated the estimated LBA model parameters of a simple choice task with the precision in temporal reproduction, estimated using a model of time estimation (Balcı & Simen, 2016; Simen, Vlasov, & Papadakis, 2016). For details of the experimental paradigm or the fitting procedures, we refer to the original publication (Miletić & van Maanen, 2019).

**Fig. 3:**
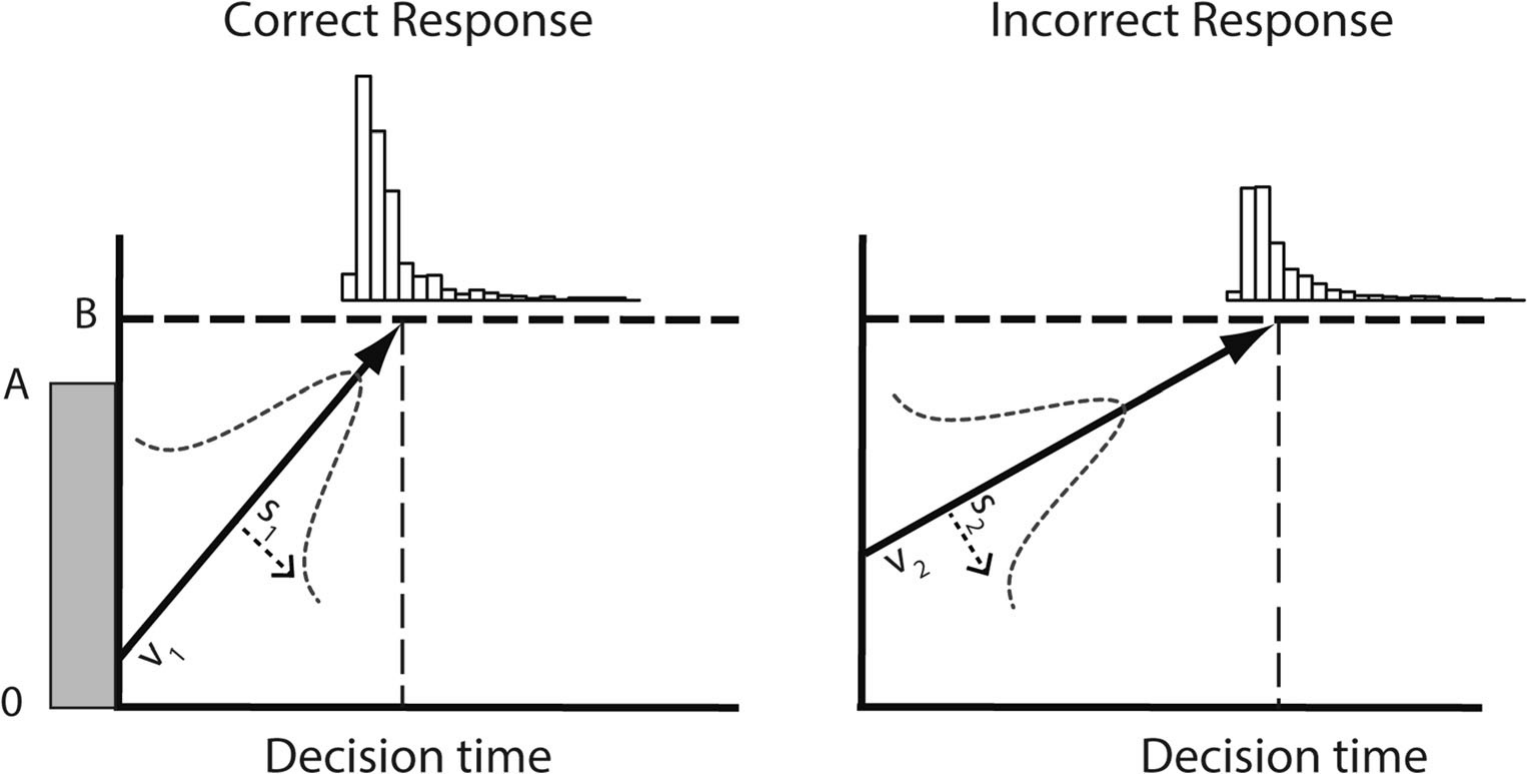
The linear ballistic accumulator model assumes that binary decisions depend on the accumulation of evidence for either choice alternative, which varies from trial to trial. The rise-to-threshold (drift rate) is characterized by a normal distribution with mean *v* and standard deviation *s.* The distance-to-threshold is characterized by a uniform distribution [*B–A, B*]. Because *v* and *s* are assumed to differ between the accumulators representing correct and incorrect choices, these parameters jointly explain the proportion of errors, as well as the observed response time distributions for different choices (shown on top)

The LBA model assumes that the noisy accumulation of evidence for a choice alternative can be approximated by a linear nonstochastic rise to a threshold value. The choice between the available alternatives follows from whichever accumulation reaches the threshold value first. Incorrect responses and response time distributions are explained by assuming variability across trials in the linear rise-to-threshold as well as the threshold value. This simple process accounts for many benchmark phenomena in (perceptual) decision making (S. D. Brown & Heathcote, 2008; Donkin & van Maanen, 2014; van Maanen, Forstmann, Keuken, Wagenmakers, & Heathcote, 2016). The standard LBA model has five parameters (see Fig. 3): The rise-to-threshold (drift rate) is represented by a normal distribution with mean *v* and standard deviation *s*. The distance to the threshold is represented by a uniform distribution [*B–A*, *B*] (i.e., *B* is the maximum threshold value, and *B–A* is the minimum, because the start point of accumulation is sampled from [0, A]; S. D. Brown & Heathcote, 2008). In addition, the LBA model assumes that the time required for perceptual processing prior to a decision stage, and the time required for executing a motor response, together result in a shift of the response time distribution (*t*_*0*_).

**Fig. 4:**
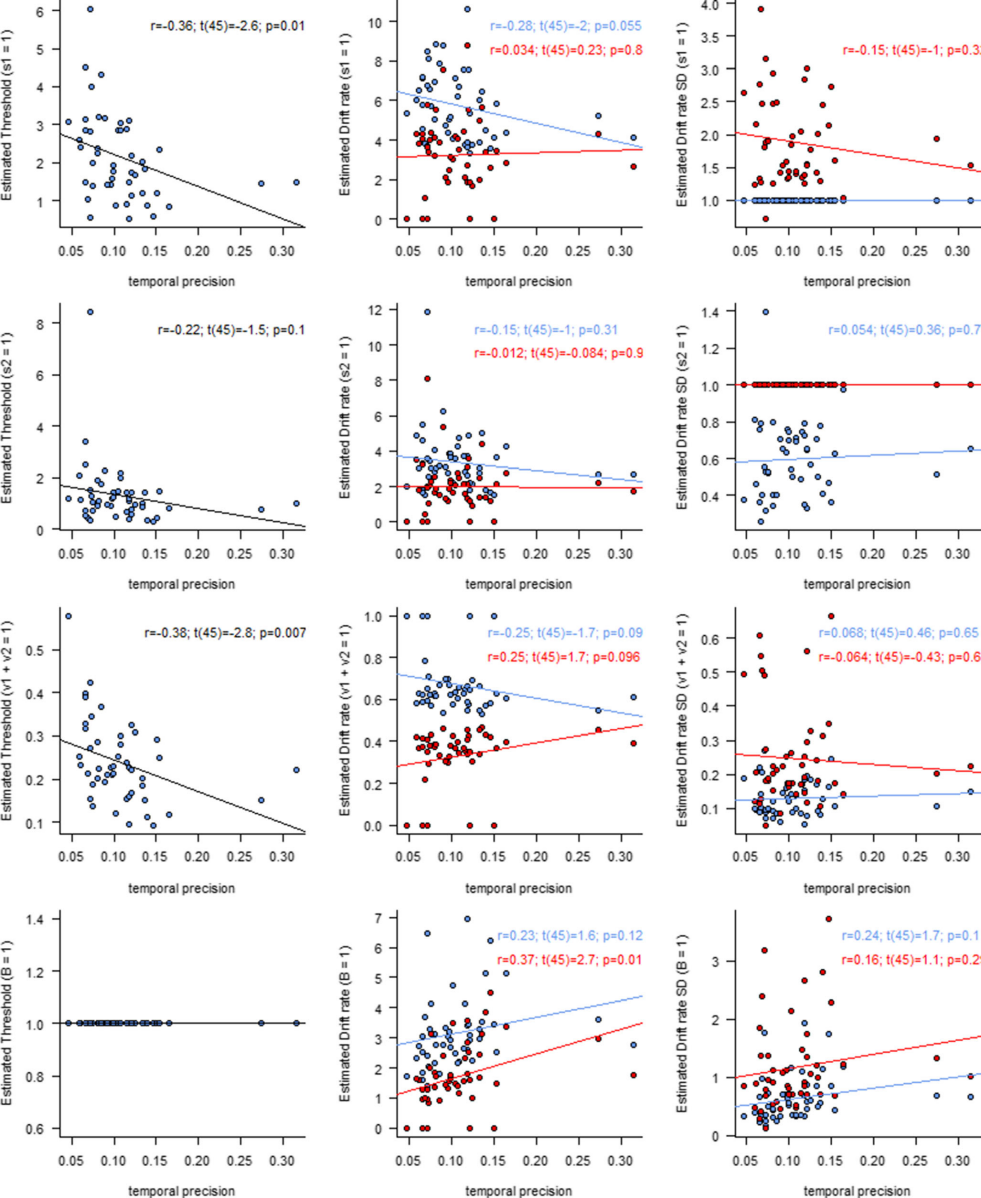
Correlations between estimated linear ballistic accumulator (LBA) model parameters and temporal precision in a secondary task. Left panels: Threshold parameter **B**. Middle panels: Mean drift rates for correct (blue, *v*_1_) and incorrect (red, *v*_2_) responses. Right panels: Standard deviation of drift rates for correct (blue, *s*_1_) and incorrect (red, *s*_2_) responses. Each row is scaled differently (indicated between brackets on the *y*-axis). The statistics are the results of correlation tests on the LBA parameter and temporal precision from Miletić and van Maanen (2019), suggestive of the different conclusions that might have been drawn if different scaling assumptions would have been made

The parameters were optimized by maximizing the summed log likelihood using the SIMPLEX algorithm (Nelder & Mead, 1965). To eliminate any implicit scaling due to boundaries of the parameter space, all parameters except *t*_*0*_ were estimated on a log scale. Note that this was done without a scaling constraint. The *t*_*0*_ parameter was estimated using a logistic transformation that mapped the range [0, min(RT)] to [−∞, ∞]. To avoid local minima, the fitting was restarted with random initial values at least 500 times.^1^ To illustrate the effect of different scaling constraints, we divided all parameters except *t*_*0*_ by various scaling constraints. Each row in Fig. 4 presents the results for a different constraint.

The top row in Fig. 4 replicates the result from Miletić and van Maanen (2019), where we assumed that the standard deviation of the winning accumulator is always the same.^2^ We found a correlation between temporal precision *m* and the threshold parameter *B*, as well as a marginally significant correlation between *m* and the mean drift rate of the winning accumulator *v*_*1*_. Of note is that we did not find correlations with the mean drift rate of the losing accumulator *v*_*2*_, nor with the standard deviation of that accumulator.^3^ Here, we assumed that the standard deviation of the winning accumulator would not vary between participants, hence the correlation of that parameter with *m* could not be verified. These results suggest that individuals who were better “timers” (indicated by a low *m*) displayed higher thresholds and possibly higher drift rates than individuals who were poorer timers (i.e., high *m*).

The second row of Fig. 4 reveals what conclusions we might have drawn, had we constrained the standard deviation of the losing accumulator (*s*_*2*_). The values on the *y*-axes represent the originally estimated parameters, but divided by the standard deviation of the losing accumulator. Although this is not necessarily identical to the result of refitting the data with the new constraint, it comes close, as differences could only be due to local minima in the parameter landscape. The interpretation of our original analysis would not be supported, although the trends in the data seem to be consistent.

The third row of Fig. 4 shows the conclusions under the constraint that the sum of drift rates is 1, which is a common assumption in LBA models (Donkin, Brown, & Heathcote, 2011). Under this assumption, the conclusion that *B* and *m* correlate can again be drawn, but not the conclusion that *v*_*1*_ and *m* correlate. Note that the correlation coefficients of the drift rates of the winning and losing accumulators are equal but of opposite sign, due to the specific scaling constraint used here (i.e., *v*_1_ + *v*_2_ = 1).

The bottom row of Fig. 4 shows what the conclusion would have been under the constraint that *B* = 1. Because we now do not allow *B* to vary across individuals, there is no association with *B* possible. Instead, *m* now predicts the *inverse* pattern of drift rates, in particular for the losing accumulator: the higher *m*, the higher *v*_*2*_. This would entail that precise timers (low *m*) display slow evidence accumulation for incorrect responses. Such a conclusion would have been meaningful had the hypothesis been that individuals who are good at time estimation also have the ability to suppress irrelevant information.

Making the scaling constraint explicit in the parametrization helps to understand the apparent ambiguity of the results. For example, if the standard deviation of the drift rate is constrained to *s*_*1*_ = 1 (see Fig. 4, top row), then the threshold parameter can be thought of as a target amount of evidence (*E*) *relative to one standard deviation of drift rate*. The unit associated with that quantity would be *E*/(*E*/*s*), since the unit of drift rates is the amount of accumulated evidence per second (note that s here refers to second, contrary to *s*_*1*_, the standard deviation of the drift rate distribution). Similarly, the unit of the mean drift rate *v* would become (*E/s*) / (*E/s*), or a unitless signal-to-noise ratio.

If, on the other hand, the threshold parameter was constrained at *B* = 1*E* (i.e., one evidence unit *E*, as in Fig. 4, bottom row), then the unit of both the mean and standard deviation of the drift rate would become (*E/s*)/*E*. Rearranging to 1/s makes clear that this can be thought of as *frequency*: it expresses the (mean and standard deviation of the) *number of threshold distances* that an accumulator covers per second. In light of the experiment we reanalyzed, this would mean that higher *m* is associated with a higher number of threshold distances covered per second, in particular for the incorrect accumulator.

Although both interpretations highlight different aspects of the cognitive processes, they are consistent with each other. The first interpretation holds that *m* correlates negatively with the threshold-to-noise ratio *b*/*s*_*1*_, but not with signal-to-noise ratio *v*_*2*_*/s*_*1*_. Hence, higher *m* indicates lower *b/s*_*1*_, but equal *v*_*2*_*/s*_*1*_. It follows that *relatively speaking*, the speed-per-threshold *v*_*2*_*/b* (for the incorrect accumulator) is higher for higher *m*. This is the second interpretation: higher *m* is associated with a higher number of threshold distances covered per second.

## Discussion

In this article, we illustrated the consequence of choosing a potentially inappropriate scaling constraint, when interpreting correlations between computational cognitive model parameters and individual differences in neurophysiological, psychological, and physical factors. In simulation, we showed that the data-generating parameters of SDT cannot be recovered, unless a scaling constraint is assumed. The choice of scaling constraint critically affects the parameter estimates, which becomes crucial for interpreting the relationship with other factors, often necessary for scientific progress. Similar findings can be observed across multiple modeling paradigms, including RL, and evidence accumulation models (DDM and LBA). In a reanalysis of a previously published experiment using the LBA model, we corroborated these findings, and found that one specific association between a model parameter and a measure taken from another task flips sign depending on the assumption adopted. That is, in the original publication we found a positive correlation between drift rate and temporal precision, suggesting that participants that were more uncertain in their temporal judgements showed faster evidence accumulation. In addition, we found a negative correlation between temporal precision and threshold, suggesting that more imprecise participants adopted lower threshold values. In a subsequent experiment we teased apart these effects, such that we remain confident about our initial conclusions (see Miletić & van Maanen, 2019).

However, assuming a different scaling constraint ultimately led to a positive correlation between the drift rate of a losing accumulator and temporal precision, suggesting that participants that were *more certain* in their temporal judgements could suppress irrelevant information. This suggestion seems plausible on the surface, and researchers might be tempted to adopt this conclusion, if they would find the scaling constraint that was used appropriate.

It is important to realize that the ambiguity in these conclusions is essentially an interpretation problem. When fitting a model while keeping one parameter constant for scaling, the estimates of all other parameters are defined relative to the scaling constraint—and thus, the interpretation of these parameters should also be understood *relative* to the scaling constraint. Different conclusions about interindividual correlations reached under different scaling assumptions may at first sight appear contradictory, but they are, in fact, consistent with one another. This becomes clear when the scaling constraint is explicitly mentioned in the parametrization. However, this interpretation step is often not made in the literature, and poses limitations on the conclusions that one can draw.

To guide the interpretation of parameter estimates, we proposed to explicitly express parameters in their respective units. Expressing parameters in their units explicates the relationships that exist between parameters. Fitting the model while keeping one parameter constant for scaling purposes *changes the units* of the estimated parameters, and consequently the interpretation. In light of this, it makes intuitive sense to interpret the evidence accumulation process implemented in LBA as a signal-to-noise ratio, although in some cases other design choices could be made (e.g., Nunez, Srinivasan, & Vandekerckhove, 2015; Nunez, Vandekerckhove, & Srinivasan, 2017). This also seems the most useful interpretation for the sensitivity and criterion of SDT.

For standard RL, the most straightforward way to explicate the relationship between *β* (the inverse temperature of the soft-max function) and *γ* (the weighting of the reward function) is to express *β* in units of 1*γ*. The alternative is also possible, if a researcher is interested in individual variation *γ*: In that case, scaling *β* to *β* = 1, means that the weighting of the reward function is expressed per 1 unit inverse temperature. More complex relations might entail when the relationship between reward and value is modelled in a nonlinear way (Ahn et al., 2008; Scholl, Kolling, Nelissen, Browning, et al., 2017; Scholl, Kolling, Nelissen, Stagg, et al., 2017; Scholl et al., 2015; Steingroever et al., 2014), making it even more important to keep track of the relationship between parameters. These examples illustrate that expressing parameters in their respective units helps in interpreting the parameter estimates, and—at least for replicability purposes—helps in specifying the model under consideration.

The contribution of the current paper is that a conclusion about the association between a cognitive model parameter and another factor is only valid under the assumption that participants indeed do not vary with respect to the scaling constraint. Unfortunately, in many papers, the scaling constraint is not mentioned, or only cursory (Tran et al., submitted). This makes conclusions about the relationships that exist between model and individual difficult to interpret, since there is no explicit justification for the scaling constraint that is applied.

Because sets of parameters can be rescaled to another parameter, it is not enough to argue that the default scaling constraint is accurate, *because* the model’s fit is good. That is, another scaling constraint results in exactly the same goodness of fit, but a different relationship with the underlying factor of interest.

Implicitly, we addressed another issue, and that is the assumption of a scaling constraint per se. In generating the data for our simulations, we made the assumption that participants differed in the values of all their parameters. In contrast, applications of the class of models under consideration here almost always assume that at least one parameter is constraint across participants for scaling purposes. This assumption may be warranted in many applications, where variability in the parameter that is selected for scaling is small and nonsystematic compared with others (cf. the SDT illustration with which we started). A solution that more closely adheres to the true variability in the data might be to hierarchically fit the cognitive model to all participants. Under the assumption that participants are hierarchically nested in a group, one could constrain only the group level parameter to a constant. This way, the scaling property is satisfied, while allowing variability around this constant on the level of participants. Using Bayesian model fitting techniques (Anders, Oravecz, & Alario, 2017; Lee & Wagenmakers, 2013), this is often implied by prior distributions of the parameters, either on the individual or group level. These prior distributions enforce the intuition that the scaling parameters are at least closely related, but allow variability if necessary. This approach might best balance the need for constraint with the reality of the data.

### Open practices statement

The SDT simulation study, the reanalyses from Miletić and van Maanen (2019), and the analyses reported in the Supplementary Materials are available online (https://osf.io/ctp9r/).

## Electronic supplementary material

ESM 1 (DOCX 2.50 mb)

ESM 2 (DOCX 329 kb

## Footnotes

1 The best fitting parameter estimates of the originally published model were also included as initial guesses.

2 Note that in the original analysis we report *response caution*, defined as *B − A*/2, rather than B. Response caution is the average distance to threshold. For the current purposes we focus on the threshold parameter instead; *m* is a parameter from a model of temporal reproduction, and is directly proportional to the coefficient of variation.

3 The original correlations did not depend on the two extreme estimates of *m*. For details, see Miletić and van Maanen (2019).

## References

[CR1] Ahn WY, Busemeyer JR, Wagenmakers EJ, Stout JC (2008). Comparison of decision learning models using the generalization criterion method. Cognitive Science.

[CR2] Anders, R., Oravecz, Z., & Alario, F. X. (2017). Improved information pooling for hierarchical cognitive models through multiple and covaried regression. *Behavior Research Methods*. doi:10.3758/s13428-017-0921-7 10.3758/s13428-017-0921-7 28699122

[CR3] Balcı F, Simen P (2016). A decision model of timing. Current Opinion in Behavioral Sciences.

[CR4] Behrens TEJ, Woolrich MW, Walton ME, Rushworth MFS (2007). Learning the value of information in an uncertain world. Nature Neuroscience.

[CR5] Berridge KC (2012). From prediction error to incentive salience: Mesolimbic computation of reward motivation. European Journal of Neuroscience.

[CR6] Brown SD, Heathcote A (2008). The simplest complete model of choice response time: Linear ballistic accumulation. Cognitive Psychology.

[CR7] Brown VM, Zhu L, Wang JM, Frueh BC, King-Casas B, Chiu PH (2018). Associability-modulated loss learning is increased in posttraumatic stress disorder. ELife.

[CR8] Daw ND, O’Doherty JP, Dayan P, Seymour B, Dolan RJ (2006). Cortical substrates for exploratory decisions in humans. Nature.

[CR9] de Lange FP, Rahnev DA, Donner TH, Lau H (2013). Prestimulus oscillatory activity over motor cortex reflects perceptual expectations. Journal of Neuroscience.

[CR10] Donkin C, Brown SD, Heathcote A (2009). The overconstraint of response time models: Rethinking the scaling problem. Psychonomic Bulletin & Review.

[CR11] Donkin C, Brown SD, Heathcote A (2011). Drawing conclusions from choice response time models: {A} tutorial. Journal of Mathematical Psychology.

[CR12] Donkin C, van Maanen L (2014). Piéron’s law is not just an artifact of the response mechanism. Journal of Mathematical Psychology.

[CR13] Forstmann, B. U., Dutilh, G., Brown, S. D., Neumann, J., von Cramon, D. Y., Ridderinkhof, K. R., & Wagenmakers, E.-J. (2008). Striatum and pre-SMA facilitate decision-making under time pressure. *Proceedings of the National Academy of Sciences of the United States of America*, *105*, 17538–17542. 10.1073/pnas.0805903105 PMC2582260 18981414

[CR14] Green DM, Swets JA (1966). *Signal detection theory and psychophysics*.

[CR15] Huys QJ, Pizzagalli DA, Bogdan R, Dayan P (2013). Mapping anhedonia onto reinforcement learning: A behavioural meta-analysis. Biology of Mood & Anxiety Disorders.

[CR16] Kaneko, Y., & Sakai, K. (2015). Dissociation in decision bias mechanism between probabilistic information and previous decision. *Frontiers in Human Neuroscience*, *9*(MAY). doi:10.3389/fnhum.2015.00261 10.3389/fnhum.2015.00261 PMC4423353 25999844

[CR17] Lebreton M, Bavard S, Daunizeau J, Palminteri S (2019). Assessing inter-individual differences with task-related functional neuroimaging. Nature Human Behaviour.

[CR18] Lee MD, Wagenmakers E-J (2013). *Bayesian modeling for cognitive science: {A} practical course*.

[CR19] Louie K, Glimcher PW (2012). Efficient coding and the neural representation of value. Annals of the New York Academy of Sciences.

[CR20] Macmillan NA, Creelman CD (2005). *Detection theory: A user’s guide*.

[CR21] Miletić S, van Maanen L (2019). Caution in decision-making under time pressure is mediated by timing ability. Cognitive Psychology.

[CR22] Moran R (2016). Thou shalt identify! The identifiability of two high-threshold models in confidence-rating recognition (and super-recognition) paradigms. Journal of Mathematical Psychology.

[CR23] Mulder, M. J., van Maanen, L., & Forstmann, B. U. (2014). Perceptual decision neurosciences—A model-based review. *Neuroscience*, *277*, 872–884. doi:10.1016/j.neuroscience.2014.07.031 10.1016/j.neuroscience.2014.07.031 25080159

[CR24] Nelder JA, Mead R (1965). A simplex method for function minimization. The Computer Journal.

[CR25] Nunez, M. D., Srinivasan, R., & Vandekerckhove, J. (2015). Individual differences in attention influence perceptual decision making. *Frontiers in Psychology*. doi:10.3389/fpsyg.2015.00018 10.3389/fpsyg.2015.00018 PMC4329506 25762974

[CR26] Nunez, M. D., Vandekerckhove, J., & Srinivasan, R. (2017). How attention influences perceptual decision making: Single-trial EEG correlates of drift-diffusion model parameters. *Journal of Mathematical Psychology*, *76B*. doi:10.1016/j.jmp.2016.03.003 10.1016/j.jmp.2016.03.003 PMC5397902 28435173

[CR27] O’Reilly JX, Mars RB (2011). Computational neuroimaging: localising Greek letters? Comment on Forstmann et al. Trends Cogn Sci.

[CR28] Palminteri S, Wyart V, Koechlin E (2017). The importance of falsification in computational cognitive modeling. Trends in Cognitive Sciences.

[CR29] Poldrack RA (2015). Is “efficiency” a useful concept in cognitive neuroscience?. Developmental Cognitive Neuroscience.

[CR30] Rahnev D, Lau H, de Lange FP (2011). Prior expectation modulates the interaction between sensory and prefrontal regions in the human brain. Journal of Neuroscience.

[CR31] Ratcliff R (1978). A theory of memory retrieval. Psychological Review.

[CR32] Ratcliff R, McKoon G (2008). The diffusion decision model: Theory and data for two-choice decision tasks. Neural Computation.

[CR33] Scholl, J., Kolling, N., Nelissen, N., Browning, M., Rushworth, M. F. S., & Harmer, C. J. (2017a). Beyond negative valence: 2-week administration of a serotonergic antidepressant enhances both reward and effort learning signals. *PLOS Biology*, *15*(2). doi:10.1371/journal.pbio.2000756 10.1371/journal.pbio.2000756 PMC5331946 28207733

[CR34] Scholl, J., Kolling, N., Nelissen, N., Stagg, C. J., Harmer, C. J., & Rushworth, M. F. S. (2017b). Excitation and inhibition in anterior cingulate predict use of past experiences. *ELife*, *6*. doi:10.7554/eLife.20365 10.7554/eLife.20365 PMC5213710 28055824

[CR35] Scholl J, Kolling N, Nelissen N, Wittmann MK, Harmer CJ, Rushworth MFS (2015). The good, the bad, and the irrelevant: Neural mechanisms of learning real and hypothetical rewards and effort. Journal of Neuroscience.

[CR36] Simen P, Vlasov K, Papadakis S (2016). Scale (in)variance in a unified diffusion model of decision making and timing. Psychological Review.

[CR37] Steingroever H, Wetzels R, Wagenmakers EJ (2014). Absolute performance of reinforcement-learning models for the Iowa gambling task. Decision.

[CR38] Sutton RS, Barto AG (2018). *Reinforcement learning: An introduction*.

[CR39] Tran, N.-H., van Maanen, L., Heathcote, A., Matzke, D. (submitted) Systematic Parameter Reviews in Cognitive Modeling: Towards Robust and Cumulative Models of Psychological Processes. Retrieved from OSF. https://www.osf.io/9ycu5/10.3389/fpsyg.2020.608287 PMC7874054 33584443

[CR40] Turner BM, Forstmann BU, Love BC, Palmeri TJ, van Maanen L (2017). Approaches to analysis in model-based cognitive neuroscience. Journal of Mathematical Psychology.

[CR41] van Maanen L, Forstmann BU, Keuken MC, Wagenmakers E-J, Heathcote A (2016). The impact of MRI scanner environment on perceptual decision making. Behavior Research Methods.

[CR42] Wilson, R. C., & Collins, A. G. E. (2019). Ten simple rules for the computational modeling of behavioral data. *ELife*, *8*. doi:10.7554/eLife.49547 10.7554/eLife.49547 PMC6879303 31769410

[CR43] Zhang J, Berridge KC, Tindell AJ, Smith KS, Aldridge JW (2009). A neural computational model of incentive salience. PLOS Computational Biology.

