# Supplementary material for: The interpretation of behavior-model correlations in unidentified cognitive models
Source: Psychon Bull Rev. 2020 Aug 6;28(2):374–83. doi: 10.3758/s13423-020-01783-y (PMC8062378; doi:10.3758/s13423-020-01783-y)
Supplement: Supplementary file 1 — (DOCX 2.50 mb) [file 13423_2020_1783_MOESM1_ESM.docx]

## Supplementary analysis 1: Linear Ballistic Accumulator

In this simulation, all LBA model parameters except the maximum threshold *B* were randomly drawn from uniform distributions, separately for each simulated participant:

$$v_{1}\sim U\left( 0.3, 0.7 \right)+v_{2}$$

$$v_{2}\sim U\left( 0.3, 0.7 \right)$$

$$s_{1}\sim U\left( 0.1, 0.5 \right)$$

$$s_{2}\sim U\left( 0.1, 0.5 \right)$$

$$A\sim U\left( 0.2, 0.3 \right)$$

$$t_{0}\sim U\left( 0.1, 0.3 \right)$$

The model thus generates choices associated with two drift rates, $v_{1}$ and $v_{2}$, where $v_{1}$ is always higher than $v_{2}$. Finally and crucially, the maximum threshold parameters were generated according to

$$B\sim U\left( 0.0, 0.5 \right)+0.05F$$

With *F* a numeric factor. Thus, we simulated a linear relationship between threshold parameters and an arbitrary factor that could represent some additional neurophysiological, psychological, or physical measurement in an experiment setting. This factor ensures that there is a systematic change in the threshold across participants, but not in any of the other parameters.

We generated 1000 trials for 20 independent participants. The high trial count ensures that standard optimization routines are able to find a set of parameters that accurately describe the data. Hence, the results below cannot be ascribed to sampling error.

We first estimated parameters for the data generated for these participants without any scaling constraint. This way, we illustrate the need for scaling constraints in the LBA model. Next, we recomputed all parameters assuming two specific scaling constraints. The first recalculation assumes that $v_{1}+v_{2}=1$, which is a very common assumption when applying the LBA model (cf. Donkin et al., 2011). In the second recomputation all parameters were adjusted such that the scaling constraint becomes $B=1$. While this may be an uncommon choice, it is not unprecedented (Nunez et al., 2015, 2017).

The parameters were optimized by maximizing the summed log likelihood using the SIMPLEX algorithm (Nelder & Mead, 1965). To eliminate any implicit scaling due to boundaries of the parameter space, all parameters except *t_0_* were estimated on a log scale. The *t_0_* parameter was estimated using a logistic transformation that mapped the range $[0, min(RT)]$ to $[-\infty,\infty]$. To avoid local minima, the fitting was restarted with random initial values at least 2000 times.

**

*Figure S1. The correlations between an underlying factor and all LBA parameters, for various scaling constraints.*

References

Donkin, C., Brown, S. D., & Heathcote, A. (2011). Drawing conclusions from choice response time models: A tutorial. *Journal of Mathematical Psychology*, *55*, 140–151.

Nelder, J. A., & Mead, R. (1965). A simplex method for function minimization. *The Computer Journal*, *7*, 308–313.

Nunez, M. D., Srinivasan, R., & Vandekerckhove, J. (2015). Individual differences in attention influence perceptual decision making. *Frontiers in Psychology*. https://doi.org/10.3389/fpsyg.2015.00018

Nunez, M. D., Vandekerckhove, J., & Srinivasan, R. (2017). How attention influences perceptual decision making: Single-trial EEG correlates of drift-diffusion model parameters. *Journal of Mathematical Psychology*, *76B*. https://doi.org/10.1016/j.jmp.2016.03.003
