# Supplementary material for: The interpretation of behavior-model correlations in unidentified cognitive models
Source: Psychon Bull Rev. 2020 Aug 6;28(2):374–83. doi: 10.3758/s13423-020-01783-y (PMC8062378; doi:10.3758/s13423-020-01783-y)
Supplement: Supplementary file 2 — (DOCX 329 kb [file 13423_2020_1783_MOESM2_ESM.docx]

## Supplementary analysis 2: Diffusion Decision Model

We generated data for 20 participants and 100s trials according to

$$v\sim U\left( 0.1, 0.5 \right)+0.05F$$

$$a\sim U\left( 0.8, 1.0 \right)$$

$$s\sim U\left( 0.8, 1.2 \right)$$

$$t_{0}\sim U\left( 0.1, 0.2 \right)$$

Between-trial variability parameters were constrained to 0, and start point of accumulation was constrained to $\frac{1}{2}a$. This simulation illustrates that the identifability issues extend to other evidence accumulation models and parameter modulations. We again used SIMPLEX to maximize the likelihood of the data for a set of model parameters (Nelder & Mead, 1965). Parameter a was log-transformed to allow it to be fit on an infinite scale (a is only identified on the positive scale). Similarly, parameter t_0_ was logistically transformed and multiplied by min(RT) to allow it to be fit on an infinite scale.

*
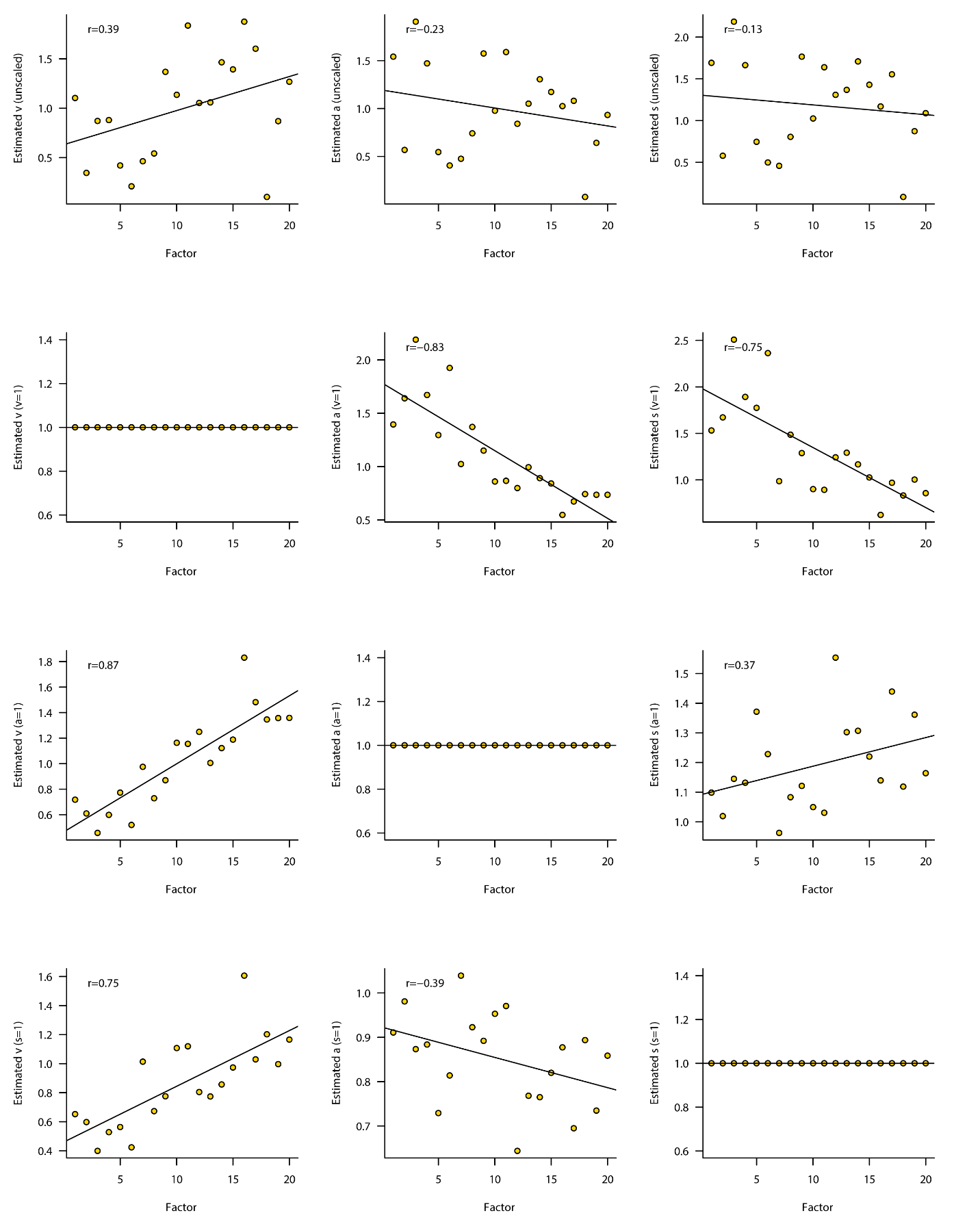
*

*Figure S2. The correlations between an underlying factor and the most important parameters, for various scaling constraints using the Diffusion Decision model.*
